# Effects of water deficit on leaves and fruit quality during the development period in tomato plant

**DOI:** 10.1002/fsn3.2160

**Published:** 2021-02-16

**Authors:** Ibtissem Medyouni, Refka Zouaoui, Emilie Rubio, Sylvie Serino, Hela Ben Ahmed, Nadia Bertin

**Affiliations:** ^1^ Laboratory of Plants Soil and Environment Interactions (LIPSE) Faculty of Sciences of Tunis University of Tunis El Manar Tunis Tunisia; ^2^ INRA—Centre d’Avignon UR1115 Plantes et Systèmes de Culture Horticoles Avignon France; ^3^ Ecology and Sylvo‐Pastoral Improvement Laboratory Water and Forests (INRGREF) National Research Institute of Rural Engineering Tunis Tunisia

**Keywords:** fruit, growth, leaves, metabolic parameters, tomato, water deficit

## Abstract

In nature, plants are often exposed to a multitude of environmental constraints that severely limit crop productivity. Water deficit is one of the factors that most affects agricultural production. The aim of this work is to evaluate the effect of water deficit on morphology, development, nutritional behavior, as well as chlorophyll fluorescence and certain important metabolic parameters (soluble sugars, organic acids, starch, carotenoid, and vitamin C) of the cultivated tomato (*Solanum lycopersicum* cv Plovdiv). In this study, the water supply was reduced by 60% compared to control conditions. The conditions of water deficit showed that the size of the different organs (leaves, fruits) was reduced. A reduction in the number, width, and length of the leaves, respectively, 9%, 36%, and 37%, then the leaf surface was also observed. Reduction of fluorescence (Fo, Fm, and Fv) and total index performance were among the other symptoms of plants with water deficiency. For fruit, we observed a significant decrease in diameter, fresh weight, and moisture content during the cell division period, the cell expansion period, and the fruit ripening period. In contrast, the composition of the Plovdiv fruit changed only during cell division and expansion phase. On the other hand, the water deficit induces an increase in the total carotenoid and vitamin C content of the fruits.. Besides, water deficit induced a reduction of fruit size, moisture content, and production dry matter during different phases of development. Decrease levels of soluble sugars and organic acid but increase in vitamin C and carotenoid content.

## Graphical abstract

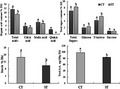

## 1 INTRODUCTION

Agriculture is exposed to frequent periods of drought and limitation of water resources what is expected to exert an adverse impact on plant growth and crop productivity (Shao et al., 2008). The Mediterranean regions are experiencing periods of intense drought, leading to the extension of arid zones (Gao & Giorgi, 2008). In view of gradually depleting irrigation water resources throughout the world, it is highly imperative to investigate the effects of water deficit in plants consuming large amounts of water like tomato (*Solanum lycopersicum*). In fact, water plays a crucial role in determining the yield of processing tomato but it is likely that water scarcity period will have to befaced in the near future. Water scarcity and increasing competition for water resources between agriculture and other sectors are forcing the search for new irrigation strategies in semi‐arid Mediterranean regions, which can reduce the consumption of irrigation water and maintain production (Costa et al., 2007). The best method to achieve the goal of improving water use efficiency (Topcu et al., 2007) is the deficit irrigation (DI), a strategy for decreasing water consumption in which crops are deliberately allowed to maintain a certain level of water deficit (WD) and yield reduction (Pereira et al., 2002). Indeed, WD effects have been extensively studied on several crops (Costa et al., 2007). The development, growth, and productivity of plants under WD conditions can be affected according to the intensity, timing, and duration of WD, as well as genotype, as observed in the tomato (*Solanum lycopersicum* L.) (Davies et al., 2000; Marjanovic et al., 2012). Xu and Zhou (2008) showed that the water deficiency affected the transpiration surface and this due to a decrease in cellular expansion, suggestion that reduction allowed the plant to better adapt and exploit the available water and reduce transporter losses, in particular by limiting the opening of the stoma (Daszkowska‐Golec & Szarejko, 2013). Further, leaf rolling is a mechanism involved in plant responses to water deficit (Puglielli et al., 2017). The water deficit has a beneficial effect on fruit quality related to higher sugar accumulation and organic acid as reported by (Ripoll et al., 2014). In preliminary studies in many species, the water deficit caused a decrease in plant growth, enhanced fruit quality (e.g., increased sugar and acid levels), and an acceleration in fruit maturation (Guichard et al., 2005; Mirás‐Avalos et al., 2013). However, the reported effects of WD on fruit quality are highly variable depending on the genotype and on the plant, fruit developmental stages and the duration of treatments (Ripoll et al., 2014). In general, in most species fruit development is devised into three phases, cell division followed by cell expansion and finally ripening period (Bertin et al., 2007). The application of WD during the division phase causes carbon deficiency and decreases the cell division and development of tomato fruit (Prudent et al., 2010). Moreover, in tomatoes (Prudent et al.,^2010^), the application of WD negatively regulate cell division and fruit tissue development in tomato. In addition, the expansion phase is a specific stage of development because it causes changes in the growth of fruit water (Schopfer, 2001). Finally, during the maturation phase, WD increases the synthesis of ethylene (Barry & Giovannoni, 2007; Fray et al., 1994). In tomato, WD showed a significant increase in fruit quality (soluble sugars, organic acids, flavors, and AsA) at the red stage compared to mature green or orange stages (Veit‐Köhler et al., 1999).

In the present study, our objectives were to study the effect of the water deficit on the tomato crop, to understand the relationship between the water deficit determined by a restriction on the amount of water and the development of the plant and the quality of the fruit, in order to allow the farmer to better manage irrigation water management and avoid problems of growth, yield, and quality of fruit.

## 2 MATERIALS AND METHODS

### 2.1 Plant material & experimental conditions

The study was performed on *S. lycopersicum. L* type genotype: Plovdiv XXIVa, is a cultivated tomato plants, Plovdiv seeds were provided by the Genetic Resource center of INRA, Avignon (France). This Genotype showed important allelic variability (SNP differences on chromosomes 3, 4, 5, 7, 8, 9, 11, and 12) (Causse et al., 2013).

The trial was conducted during winter 2014 in a glasshouse located near Avignon, France. 40 plants were grown in pots (Plant/pots) filled with compost (substrate 460, Klasmann, Champety, France) distributed in two rows (control and stressed plants) at a density of 1.3 plant m^−2^ and 20 plants were actually used for the experiment. Plants were supplied daily with a nutrient solution (Liquoplant Rose, Plantin, Courthézon, France) diluted between 0.4 ‰ and 0.8 ‰ according to the plant development stage, which corresponds to an average electroconductivity of 1.8 mS cm^−1^ for the whole period. Flowers were pollinated three times a week using an electrical bee. Day–night temperature control was set at 25–15 ^◦^C. Over the whole trial period, the air temperature and relative humidity remained relatively stable (on average the day temperature ranged between 20.4 and 24.5 ^◦^C, the night temperature between 15.1 and 19.7 ^◦^C, and the air humidity between 56% and 72%). Control plants were irrigated, according to current practices, in order to maintain soil humidity and drainage around 70% (maximum water retention capacity of the substrate) and 15%, respectively. Soil humidity was measured every two days in all pots using water content sensors (WCM‐control, Grodan, Roermond he Netherlands). During the study, water supply was reduced by 60% compared to control conditions. Preliminary experiments demonstrated that this level or irrigation induced a moderate water stress based on several plant indicators (leaf conductance, stem and leaf water potentials, and the specific leaf area (SLA). Moreover, it was observed that four days were necessary to reach stable soil humidity after the beginning of water restriction in the conditions of our trial (Ripoll et al., 2016). Therefore, treatments were applied four days before the beginning of development phase. The substrate water content was measured in all pots every day and maintained around 25% for treated plants, and more than 60% for control plants.

### 2.2 The measured parameters

#### 2.2.1 Morphological parameter

Plant leaf number, width, and leaf length were measured every 3 days out of 10 plants per treatment. Mature leaves nonsenescent, which were initiated during the WD treatments, were harvested on each plant and their specific leaf area. was measured. Leaf area was measured with a Planimeter (Li‐ 3,100 C Area Meter, Li‐Cor, Lincoln, NE, USA) and leaf dry weight was measured after seven days at 70 ^◦^C in a ventilated oven. All fruit measurements were made on fruits harvested at different stages of fruit development. Ten fruits per treatment were served to measure. Fruit size, fresh weight, and water content were measured immediately after harvest.

#### 2.2.2 Physiological parameter

Fluorescence Chlorophyll parameters (Table 1) were measured on dark‐adapted leaves (30 min.) using a fluorimeter (HANDY‐PEA, Hansatech, King's Lynn, UK). Dark‐adaptation allowed the PSII electron acceptor pool to be gradually re‐oxidized to a point where all PSII reaction centers are capable of undertaking photochemistry. Measurements were carried out with an induction period of 1 s and leaves were illuminated to a light level of 3,000 μmol photons m^− 2^ s^− 1^. The measurements were carried out on nonsenescent mature leaves, at around 11 a.m. Fluorescence was measured weekly during the entire culture period. For the measurement of cations, plant material was dried at 80°C and digested with nitric acid [1% (v/v) nitric acid (HNO_3_) according to the method of Wolf (1982).

**TABLE 1:** Chlorophyll fluorescence parameters

*F_o_*	The first reliable fluorescence value after the onset of actinic illumination
*F_m_*	Maximum value under saturating illumination
*F_v_*	Maximum variable Chlorophyll fluorescence
*Fo/Fm*	A parameter related to changes in heat dissipation in the photosystem II antenna
*Fv/Fm*	The maximum photochemical efficiency of light harvesting in PSII
*Fv/Fo*	Quantum yield of primary PSII photochemistry, represents the contribution to the PI of the light reactions for primary photochemistry
V_J_	The fluorescence at J step (2 ms), F2ms
V_I_	The fluorescence at I step
ABS/RC	Specific fluxes or specific activities
DI_0_/RC	
TR_0_/RC	
ET_0_/RC	
PI_ABS_	Performance index
PI_T_	

#### 2.2.3 Mineral analysis

For the measurement of cations, plant material was dried at 80°C and digested with nitric acid [1% (v/v) nitric acid (HNO3)] according to the method of Wolf (1982). K^+^, Ca ^2+^, and Mg^2+^ were analyzed by flame emission using a spectrophotometer (Eppendorf Geratebau Netherler).

#### 2.2.4 Biochemical parameter

Fruits and leaves were frozen in liquid nitrogen and kept at − 80◦C prior to biochemical analysis soluble sugars, starch, organic acids, and carotenoids. Soluble sugars (glucose, fructose, and sucrose) and organic acids (i.e., citric acid, malic acid, and quinic acid) were extracted according to the method described by Gomez et al. (2002) and analyzed by HPLC (Waters 410, Part WAT070390, Milford, U. S. A.). Ascorbic acid content was measured according to the method described by Stevens et al. (2006), and the absorbance was read at 550 nm using a Multiscan Ascent MP reader (Labsystems, Thermo Fisher Scientific, Courtaboeuf, France). Carotenoids (i.e., lycopene, phytoene, beta‐carotene, and lutein) were extracted according to the method described by Serino et al. (2009) and assayed by HPLC with a UV–vis detector (UV6000LP, Thermo Separation Products, Riviera Beach, U. S. A.).

### 2.3 Statistical analysis

Statistical analyzes were performed using the SPSS for Windows software, version 21.0. Mean values and standard error (SE) were obtained from at least 10 measurements for physiological parameters (DW, WC, leaf area, number, width, and length of leaves) and 5 measurements for biochemical parameters. A P value under 5% was considered statistically significant. Duncan's multiple range test was used to perform means’ comparisons.

## 3 RESULTS

### 3.1 Water deficit effect on leaf morphology

**FIGURE 1:**
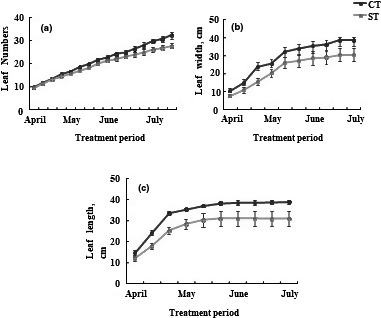
(a) Leaf number; (b) leaf width; (c) leaf length, of the leaves of tomato plants Solanum lycopersicum (cv. Plovdiv) cultivated in the presence of water (CT: control treatment) or under water restriction conditions (ST: stressed treatment), measurements were taken from the vegetative stage to the maturation stage, about 10 plants per treatment. Bar indicated standard error (*p* =.05)

After three months, the results showed that water deficit treatment decreased number of leaves (Figure 1a). The number of stressed leaves was reduced by approximately 9% compared to control leaves. Our results showed that water deficit treatment decreased leaf length and leaf width as compared to control plants (Figure 1b, c), Our results showed that treatment of water deficit decreased the length and width of leaves compared to control plants (Figure 1b, c), this reduction is respectively by of 21% and 20%.

**FIGURE 2:**
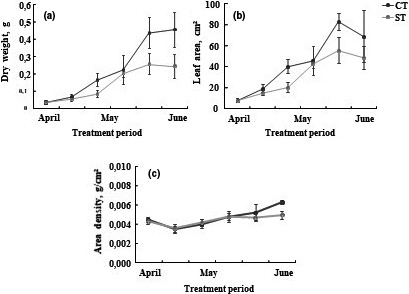
(a) Dry weight, (b) leaf area, (c) area density, of the leaves of tomato plants Solanum lycopersicum (cv. Plovdiv) cultivated in the presence of water (CT: control treatment) or under water restriction conditions (ST: stressed treatment), measurements were taken from the vegetative stage to the maturation stage, about 10 plants per treatment. Bar indicated standard error (*p* =.05)

Our results showed that water deficit decreased dry weight for tomato as compared to control plants (Figure 2 a). The dry weight was reduced by approximately 36% compared to control.

The leaf area and the area density of tomato plants were reduced not significantly with water deficit. In fact, leaf area was reduced by approximately 28% compared to control leaf area. In the same way, area density of stressed plants was reduced about 37% than control plants (Figure 2 b, c).

### 3.2 Water deficit effect on leaf physiology

#### 3.2.1 Fluorescence parameters

Illumination of a dark‐adapted leaf induces characteristic changes in fluorescence intensity. F_o_ and F_M_ respectively represent the intensity of the minimum fluorescence (all the reaction centers are oxidized or open) and maximum (all the reaction centers are reduced or closed). The rapid increase in chlorophyll fluorescence yield between F_O_ and F_M_ during the first second of intense illumination was used to analyze electron transport in the PSII. Chlorophyll fluorescence was measured on the leaf at stages10. Analysis of variance showed that irrigation regime, time significantly affected all measured traits. The intensity of the minimum F_O_ and maximum fluorescence F_M_ estimated, in tomato seedlings subjected to water deficit decreases compared with control plants. The highest Fv was decreased to plants subjected to water deficit, The FV was reduced by approximately 6% compared to control. Also, the Fv/Fm ratio significantly decreased with increasing water deficit stress severity during all the sampling times (Table 2). After a period of water deficit, a significant increase was observed for Vi and Vj and no difference compared to the control was observed for the parameters of the activity of the flows ABS/RC, DI_0_/RC, TR_0_/RC, ET_0_/RC. The water deficit induced a significant increase in IP_ABS_, this increase was about 12% compared to the witness. Under a water deficit, Plovdiv tomatoes showed a significant decrease in PI_T_ and it varied between 4.067 (control plants) and 2.409 (stressed plant).

**TABLE 2:** Variation of F_O_, F_M_, F_V_, Fluorescence Reports, Specific energy flusces and Performance index of the leaves of tomato plants *Solanum lycopersicum* ***(*** *cv*. Plovdiv) cultivated in the presence of water (CT: control treatment) or under water restriction conditions (ST: stressed treatment), measurements were taken from the vegetative stage to the maturation stage, about 10 plants per treatment

Fluorescence parametres			
F_o_		F_m_	F_v_
CT	6,189.35 ± 162.74a	34,950.14 ± 1,015.30a	28,953.17 ± 1,015.46a
ST	5,996.97 ± 117.72a	33,335.66 ± 905.45b	27,146.32 ± 1,009.32b

Fluorescence reports					
Fo/Fm		Fv/Fm	Fv/Fo	V_J_	V_I_
CT	0.175 ± 0.006a	0.825 ± 0.006a	4.865 ± 0.180a	0.432 ± 0.019a	0.808 ± 0.016a
ST	0.191 ± 0.010b	0.808 ± 0.010b	4.492 ± 0.219b	0.392 ± 0.019b	0.754 ± 0.024b

Specific energy flusces				
ABS/RC		DI_0_/RC	TR_0_/RC	ET_0_/RC
CT	1.664 ± 0.058a	0.296 ± 0.0231a	1.368 ± 0.039a	0.773 ± 0.033a
ST	1.669 ± 0.104a	0.338 ± 0.046a	1.330 ± 0.061a	0.807 ± 0.053a

Performance index		
PI_ABS_		PI_T_
CT	4.382 ± 0.395a	4.067 ± 1.479a
ST	5.029 ± 0.501b	2.409 ± 0.426b

Note

Bar indicates standard error (*p* = .05). Same letter above the bars denotes that the difference between means were not significant.

#### 3.2.2 Mineral analysis

**FIGURE 3:**
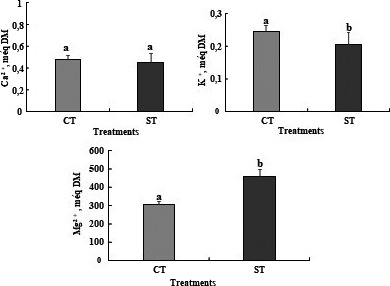
Ca^2+^, K^+^, and Mg^2+^ contents in the leaves of tomato plants *Solanum lycopersicum* (cv. Plovdiv) cultivated in the presence of water (CT: control treatment) or under water restriction conditions (ST: stressed treatment). Bar indicates standard error (*p* = .05)

The water deficit decreased the mineral content in leaves of tomato plants. Content of K^+^ and Ca^2+^ were decreased by water deficit, which suggests a deficit in providing the plants with essential ions to the growth. The water deficit reduced a K^+^ and Ca^2+^ concentration in leaves respectively by, 16% and 5% (Figure 3). However, water deficit induced an increase in Mg^2+^ content compared of control plants (Figure 0.3).

#### 3.2.3 Water deficit effect on leaves biochemical parameters

**FIGURE 4:**
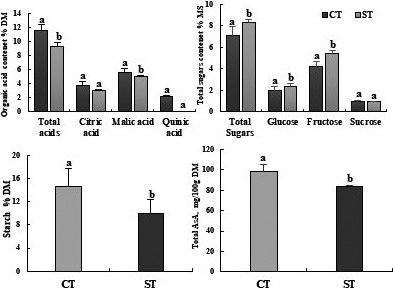
Soluble sugars and acid organic content in leaves of tomato plants Solanum lycopersicum (cv. Plovdiv) cultivated in the presence of water (CT: control treatment) or under water restriction conditions (ST: stressed treatment), Bar indicates standard error (*p* = 005)

After three months of irrigation treatment, different metabolite contents: soluble sugars, organic acids, and ascorbic acid (AsA) were measured in leaves of tomato plants Plovdiv (Figure 4). The level of acids in leaf decreased significantly with 19%, in detail the citric acid decreased with 19% and the malic acid decreased with 18% as compared of control plants. Also, the water deficit increased significantly the leaf sugar level sugar: glucose 17% and fructose 22% as compared of control plants. Furthermore, the water deficit was decreased significantly the content starch with 31% compared to control leaves. Total AsA content of leaves decreased below that of the controls, the water deficit treatment, significantly decreased the AsA by 15% compared to control.

### 3.3 Water deficit effect on fruit morphology

Fruit diameter, fresh weight, dry weight, and water content were affected by the WD treatments (Table 3). The significant increase in fresh weight, fruit diameter, dry weight, and water content was observed during the cell division phase respectively (22%, 47%, 50%, and 53% respectively). During the phase of cell expansion of the fruits. Only a significant increase in fresh weight and water content (21% and 54% respectively by). On the contrary, the treatments did not impact the diameter, height, and dry weight. In Plovdiv fruits, during the maturation phase diameter, height, fresh weight, and dry weight had not affected by water deficit. Whereas the water content decreased about 31% compared to control.

**TABLE 3:** Relative difference in fruits (Diameter, height, fresh weight, dry weight and water content) of tomato plants *Solanum lycopersicum* (cv. Plovdiv) cultivated in the presence of water (CT: control treatment) or under water restriction conditions (ST: stressed treatment) during the cell division period, the cell expansion period, and the fruit maturation period, respectively

	Phase of cell division	Cell expansion phase	Maturation phase
Diameter (mm)			
CT	6.17 ± 0.91a	31.73 ± 4.21a	37.82 ± 1.55a
ST	4.76 ± 1.07b	28.73 ± 3.71a	34.82 ± 1.13b
Height (mm)			
CT	7.12 ± 1.12a	32.4 ± 3.38a	38.36 ± 3.44a
ST	5.78 ± 1.01b	31.76 ± 2.63a	35.34 ± 2.09b
Fresh weight (g)			
CT	0.17 ± 0.07a	19.99 ± 6.77a	31.6 ± 3.71a
ST	0.09 ± 0.04b	15.65 ± 4.90b	25.88 ± 3.39b
Dry weight (g)			
CT	0.02 ± 0.01a	1.52 ± 0.53a	2.59 ± 0.37a
ST	0.01 ± 0.005b	1.47 ± 0.54a	2.49 ± 0.21a
Water content			
CT	8.36 ± 0.54a	21.56 ± 6.63a	13.78 ± 1.83a
ST	4.43 ± 0.81b	9.83 ± 1.20b	9.39 ± 1.21b

Note

Bar indicates standard error (*p* = .05). Same letter above the bars denotes that the difference between means were not significant.

### 3.4 Water deficit effect on fruit physiology

**FIGURE 5:**
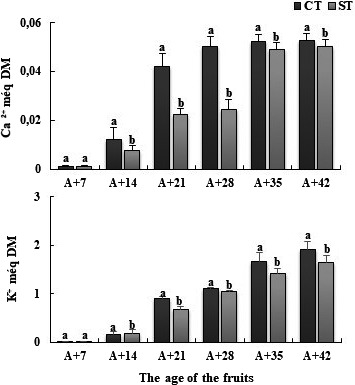
Ca^2+^ and K^+^ contents in the fruit of tomato plants Solanum lycopersicum (cv. Plovdiv) cultivated in the presence of water (CT: control treatment) or under water restriction conditions (ST: stressed treatment), Bar indicates standard error (*p* =.05)

Whatever the ages of fruits, the deficit induced a significantly decrease in K^+^ content as compared to control. In addition, the decrease of Ca^2+^ content is important in 21‐ and 28‐day‐old fruits, noted that this concentration is 0.04 meq for control fruit ages 21 days while it is only 0.02 m eq in fruit stresses (Figure 4), the reduction was about 46%. for fruits aged 28 days the concentration is 0.05 m eq for control fruit and 0.02 meq for stressed fruits, the reduction was about 51%. The water deficit treatment significantly decreased K^+^ content in fruit of tomatoes. The water deficit treatment significantly decreased K^+^ content in fruit of tomatoes. The water deficit treatment significantly decreased K^+^ content in fruit of tomatoes. The water deficit treatment significantly decreased K^+^ content in fruit of tomatoes. This decrease of K^+^ is important in 35‐ and 42‐day‐old fruits, for example, the reduction of aged fruits 35 days K^+^ content was about 1.66 m eq for control fruits and 1.41 meq for stressed fruits, the reduction was about 15% (Figure 5).

### 3.5 Effect of water deficit on the biochemical parameters of the fruits

Total sugars, total acid, AsA, and carotenoid were measured at cell expansion phase and maturation phase showed a general decrease in treated plants as compared to control plants. Contrasting responses were observed between the two ages of the fruits (Table 4).

**TABLE 4:** Relative differences in metabolite contents (soluble sugars, organic acids, ascorbic acid (AsA) and carotenoids) in the fruit red and green of tomato plants *Solanum lycopersicum* (cv. Plovdiv) cultivated in the presence of water (CT: control treatment) or under water restriction conditions (ST: stressed treatment), Bar indicates standard error (*p* = .05)

	Green fruits		Red fruits	
	CT	ST	CT	ST
Glucose	13.12 ± 2.48a	10.18 ± 1.21b	21.21 ± 1.3a	20.89 ± 1.27a
Fructose	11.16 ± 3.17a	9.03 ± 0.59b	18.58 ± 1.11a	17.17 ± 1.56a
Sucrose	0.91 ± 0.17a	1.20 ± 0.17b	1.12 ± 0.08a	2.47 ± 0.40b
Total sugars	25.02 ± 5.81a	20.42 ± 1.80b	40.91 ± 2.37a	40.32 ± 2.54a
Citric acid	2.75 ± 0.53a	3.04 ± 1.1a	3.38 ± 0.31a	3.65 ± 0.12a
Malic acid	1.88 ± 0.34a	1.61 ± 0.53a	2.53 ± 0.29 a	1.69 ± 0.35b
Total acid	4.00 ± 3.11a	4.65 ± 1.62a	5.90 ± 0.54a	5.34 ± 0.23a
Lycopene	3.16 ± 0.43a	4.17 ± 1.21b	0.37 ± 0.05a	0.32 ± 0.05a
Beta‐carotene	–	–	22.65 ± 8.82a	61.51 ± 8.82b
Phytoene	2.00 ± 0.15a	2.62 ± 0.29b	1.82 ± 0.24a	2.82 ± 0.26b
Lutein	–	–	7.12 ± 3.22a	27.90 ± 4.94b
Total carotenoids	5.16 ± 0.84a	6.78 ± 1.99b	31.12 ± 0.18a	92.23 ± 0.58b
Total AsA	18.09 ± 5.70a	22.80 ± 3.79b	13.24 ± 1.40a	21.36 ± 1.70b

Same letter above the bars denotes that the difference between means were not significant.

In fact, for the green fruit, content of fructose (19%), and glucose (22%) were decreased significantly respectively 19%, 22% compared to control plants. Moreover, green fruit increased citric acid (10%), and thus total acid (25%) as compared to their in control plants. Concerning the ascorbic acid (AsA) showed higher content of vitamin C (20%) then those of control plant. Finally, the lycopene (24%), phytoene (11%) and thus total carotenoids (23%) were increased in green fruit after water restriction treatment compared to control plants.

The sugar content was reduced in the red fruits compared to the control plants. It is found that sucrose is the more decreased sugars. The reductions are significant about 54% compared to controls. The level of organic acid in red fruits decreased with 9%, the malic acid decreased significantly more is about 33% compared to citric acid. The water deficit increased the carotenoid content in red fruits of tomato plants. In contrast to green fruits, Beta‐carotene and Lutein were present in red fruits, with significantly higher values compared to control fruit. This reduction is respectively 63% and 73%. Which suggesting the water deficit increasing the synthesis of carotenoids in mature fruits. Moreover, in the presence of water deficit, the Vitamin C was accumulated in red fruits less than green fruits. This significant increase is of the order of 38% compared to control red fruit.

## 4 DISCUSSION

Water deficit causes numerous disturbances of many functions of the cell and the whole plant. Sensing mechanisms, yet to be identified, initiate the responses to water deficit, which occur at the molecular, metabolic, cellular, physiological, and developmental level. In the present work, we will study the effect of water deficit on the tomato crop to understand the relationship between the water deficit determined by a restriction on the amount of water and the development of the plant and the quality of the fruit. Leaf is an essential organ because it has an important role in the regulation of respiration, in the synthesis of organic matter contributing to nutrition and therefore to the growth of the plant. The response of the plant to environmental conditions and more particularly to the water deficit, which is the most limiting stress of growth, has an impact on the leaf of tomato structure. It differs according to the nature and the duration of the stress (Anjum et al., 2011). The present study showed that after 3 months, water deprivation decreased number, widths, and length of leaves (Figure 1). With regard to the effect of WD on leaf area, the treatment resulted in a decrease compared to control. However, this reduction was not significant. This decrease may be due to a decline in the epidermal cell mitotic activity which results in a reduction in the total number of leaf cells (Farooq et al. 2009). Also, similar results were showed in *Pennisctum glaucum* L (Kusaka et al., 2005), *Phaseolus Vulgaris*, *Sesbania aculeate* (Ashraf and Iram, 2005) and sesame (Hassanzadeh et al., 2009). JIP parameters are increasingly recognized by plant biologists, in addition to other indicators of physiological status (Chen et al., 2014; Wituszynska et al., 2015). Fluorescence parameters F_O_, Fm, and Fv are affected by the water deficit. A general decrease in fluorescence ratios (Fo / Fm, Fv / Fm) to a greater extent, similar results have been observed in Ripoll et al., 2016. On the other hand, Specific energy fluxes were hardly changed by the water deficit, and PI_ABS_ performance indices were not reduced compared to the control, Ripoll et al., (2016) showed that PI_ABS_ decreased in LA1420 plants did not decrease in similar plants with severe water deficit.

The presence of water deficit disrupts essential mineral nutrition of tomato leaves (Dugo, 2002). In fact, the water deficit induces a decrease in the concentrations of K^+^ and Ca ^2+^ and accumulation of Mg^2+^ in the leaves. El Fakhri el al., (2011) showed that water deficit increased the concentration of K^+^ and Ca^2+^ ions in the leaves. The water deficit limits the feeding of cereals to essential nutrients, the stressed plants accumulate Na^+^ and Cl^‐^ in their organs (leaves and roots) by severely limiting the supply of K^+^ and Ca^2+^ (El Fakhri el al.,2011). Mg^2+^ content increased in the leaves of dehydrated plants, while concentrations of N and P were maintained which could partially protect photosynthetic machinery (Mahouachi, 2009).

Drought induces metabolic changes in plant, water deficit can elicit accumulations of nutrients in tomato plants such as increased levels of free sugars (Goldschmidt & Huber, 1992). Soluble sugars and organic acid mainly malic acid and citric acid are major osmotic compounds (Ripoll et al., 2014). These sugars accumulated in stressful conditions and can be simple sugars (glucose, fructose), alcohol sugars (glycerol, sorbitol, pinnitol) or complex sugars (trehalose raffinose and fructans) (Bohnert & Jensen, 1996). In the present study the water deficit caused an increase in soluble sugars in leaves compared to control (Figure 4). Indeed, the accumulation of organic solutes (sugars,) seems to play a very important role in maintaining a turgor pressure by a decrease in water potential, it is a method of adjustment of the osmotic potential (Slamane, 2015). This type of tolerance allowed the plant to perform its physiological functions normally in spite of a degradation of its internal hydric state under the effect of drought. This increase is in fact a parameter of adaptation to the conditions of water deficit, making it possible to constitute a guarantee for the maintenance of a high cell integrity (Bensalem, 1993). Similar Girona et al., (2004) and Ripoll et al. (2016) reported that water deprivation increases soluble sugars during the cell expansion phase in peach and tomato. In plant cells, the starch is present as a mixture of two polysaccharides: amylose and amylopectin. In the present study, the water deficit decreased the starch content in the leaves of tomato (Figure 4). The diminution could be explained by a decrease in photosynthetic activity and stomatal closure on plants (Teulat et al., 1997).

Vitamin C is present in all plants and compartments as it plays important roles in plants. It is very essential for plant growth and development. In leaves, vitamin C content is height in young leaves still growing and decreases in nature and prosenescent leaves as reported by Bulley et al. (2009), Li et al. (2010). According to Wang et al. (2012), tolerance to water deficit was found conflated with AsA accumulation, which plays an important role in ROS detoxification. In the present study, water privation for three months, decreased the vitamin C content in the leaves of treated plants as control (Figure 4), this may be confused with the hypothesis that AsA is not limited by photosynthesis or sugar availability, but because of environmental condition (Gautier et al., 2008). Similar research showed that water deficit increased Vitamin C levels in the leaves, suggesting a positive interaction of ascorbic acid in the protection of chloroplasts and other cell compartments (Zhu, 2001). On leaves, vitamin C content is high in young leaves still growing and decreases in mature and presenescent leaves (Bulley et al., 2009; Chen et al., 2003; Li et al., 2010).

Fruit fresh and dry masses, diameter, height, water content, mineral nutrition, concentration in soluble sugars, organic acids, AsA, and carotenoids were measured during the development phases of the tomato fruit (Phase of cell division, cell expansion phase, and maturation phase). Several studies reported the negative effect of water deficit on cell division on many species such as grape berries (*Vitis vinferal*), (McCarthy et al., 2002; Ojeda et al., 2001) and olives (*Olea europaea* L), (Gucci et al., 2009). In the present study, the water deprivation for three months decreased the fruit diameter, height, and water content respectively as compared to control tomato plants. Similar work (Wang & Gartung, 2010) reported that water deficit decreased fruits seize, weight, and water content, indeed, that fruit decreased proportionally to the intensity of WD in peach trees. Also, in our results, we found that WD caused a reduction in the weight of fruit as well the dry matter of tomato fruit respectively by about 43% and 26% as compared to control tomato plants (Table 3). The application of the water deficit throughout the tomato fruit development phase (cell division phase, cell expansion phase, and the maturation phase) induces a decrease in the concentration of K^+^ and Ca^2+^. The effect of WD on the mineral composition of tomato fruits remains poorly documented in literature. Soluble sugars and organic acids (primarily malic and citric acids) are major osmotic compounds that accumulate fleshy fruits (Ripoll et al., 2014). Acids citric, malic acids which were found in fresh tomato fruit, promotes gastric secretion, acts as a blood purifier and works as intestinal antiseptic (Pruthi, 1993), and determine taste and represent more than half of the total dry matter in tomatoes. Generally, the predominant organic acid in ripe fruits varies between species (Etienne et al., 2013). The sugars that have accumulated in stressful conditions and can be simple sugars (glucose, fructose), alcohol sugars (glycerol, sorbitol, pinnitol) or complex sugars (trehalose raffinose and fructans) (Bohnert & Jensen, 1996,). In the present study, fruit composition in soluble sugars, organic acids, carotenoids and vitamin C were determined for green and red fruits. The water deficit was at the origin of the decrease in the accumulation of soluble sugars and an increase in the accumulation of organic acids in green fruits (Table 4). Similar studies on tomatoes have shown a decrease in sugars under the conditions of WD applied during the cell division phase (Ripoll et al., 2016). Plant tissues accumulate organic acids (Hummel et al., 2010) to reduce their osmotic potential and prevent the reduction of cell turgor pressure. The accumulation of solutes or osmolytes helps maintain an osmotic balance at the cell level under dehydration conditions (Bray et al., 2000). Vitamin C is present in all plants and compartments as it plays important roles in plants. It is very essential for plant growth and development. In our study, the water deficit increased the vitamin C content of the green and red fruit of the treated plant as a control. Application of water stress during the ripening phase increased vitamin C levels (Table 4). The results suggest a positive interaction of ascorbic acid in cell compartments (Zhu, 2001). Tolerance to water deficit is correlated with AsA accumulation, which plays an important role in ROS (reactive oxygen species) detoxification (Wang et al., 2012). Indeed, vitamin C is a major antioxidant for the plant, capable of neutralizing the active forms of oxygen. These results were similar to those of Ripoll et al. (2016) who found that the application of water stress during the maturation phase increased the vitamin C levels. The results suggest a positive interaction of ascorbic acid in cell compartments (Zhu, 2001). In the plant, carotenoids, secondary pigments collecting light energy, antioxidants and precursors of hormones (abscisic acid and strigolactones), contribute to several major physiological functions and contribute to the adaptation of the plant to micro climatic variations (Gomez‐Roldan et al., 2008). In this study, the application of water deficiency during the ripening phase affected the composition of Plovdiv fruits. The composition of fruits in organic acid and soluble sugars has been barely modified by the water deficit due to a decrease in malic acid and glucose and fructose, similar results have been reported by (Ripoll, et al. 2016). In the plant, carotenoids, secondary pigments that collect light energy, antioxidants, and hormone precursors (abscisic acid and strigolactones), contribute to several major physiological functions and contribute to the adaptation of the plant to microclimatic variations (Gomez‐Roldan et al., 2008). In this study, water deficit increased carotenoid content (Table 4) for green and red fruits. Similar results were observed in tomatoes (Ripoll et al. 2016). In addition, De Pascale et al. (2007) showed that carotenoids and ascorbic acid were involved in the detoxification of reactive oxygen species that accumulate in response to different constraints. Variations in carotenoids and AsA would result from stress‐induced cellular redox changes (Fanciullino et al., 2014).

## 5 CONCLUSION

In the present study, water deficit treatment induced independent response pathways in leaves and fruits. Reduction of dry matter production, reduction of leaf area to reduce the amount of water used. water deficit reduces the accumulation of mineral ions in the leaves. Significant decrease in the levels of organic aids and vitamin C in the leaves. At the fruit level, water stress induces a reduction in fruit size, moisture content and dry matter production during the three phases of fruit development. The nutritional quality of the fruit is not affected by the water deficit. In general, soluble sugars and organic acids are stable. The nutritional quality associated with carotenoids and AsA levels could be positively affected. The action of the water deficit on tomato varieties (Plovdiv) is negative. We have also noted a certain regulation of growth to reduce the amount of water used. In addition, moderate moisture defect affects the nutritional distribution of leaves and fruits. In addition, the water deficit improves the production of carotenoids and vitamin C in fruits.

## CONFLICT OF INTEREST

The authors declare that the research was conducted in the absence of any commercial or relationships that could lead to a conflict of interest.
